# Correcting myths about stress and memory: a commentary on Pezdek and Reisberg, 2022

**DOI:** 10.3389/fpsyg.2023.1078021

**Published:** 2023-04-17

**Authors:** Carey Marr, Henry Otgaar, Conny W. E. M. Quaedflieg, Melanie Sauerland, Lorraine Hope

**Affiliations:** ^1^School of Clinical Medicine, Discipline of Psychiatry and Mental Health, University of New South Wales, Sydney, NSW, Australia; ^2^Department of Clinical Psychological Science, Maastricht University, Maastricht, Netherlands; ^3^Leuven Institute of Criminology, Catholic University of Leuven, Leuven, Belgium; ^4^Department of Neuropsychology and Psychopharmacology, Maastricht University, Maastricht, Netherlands; ^5^Department of Psychology, University of Portsmouth, Portsmouth, United Kingdom

**Keywords:** acute stress, eyewitness memory, psychological myths, emotional memory, forensic settings

## Introduction

The question of how acute stress might affect memory has applied value because witnesses, victims, and perpetrators often report experiencing stress or associated emotions (e.g., fear) during a crime. They might also experience acute stress when they are interviewed by the police. It is therefore important that legal professionals and memory scientists, particularly those acting as expert witnesses, can rely on evidence-based knowledge concerning the acute effects of stress on memory.^1^ Pezdek and Reisberg (2022) recently published an article aimed at debunking six psychological myths about evidence in the legal system. In their article, they argued that the idea that high stress improves the accuracy of memory is a myth (Myth #2). We take issue with this assertion on the basis that such a conclusion is not empirically warranted and does not accurately reflect the current state of research. In this commentary, we lend some critical nuance regarding the complex stress-memory relationship in eyewitness contexts.

## Discussion

In their article, Pezdek and Reisberg noted that they “focused on myths of which the contrary evidence seems particularly clear” (p. 144) and that the evidence they provided showed that “these widely held beliefs are (at least) without basis and, in many cases, flatly false” (p. 143). Although research on stress and memory has been ongoing over several decades, evidence or consensus on this topic is not as clear-cut as suggested. In a recent survey of 73 memory experts, Marr et al. (2021a) showed that 95% of eyewitness experts and 81% of fundamental memory experts generally agreed that “*Very high levels of stress impair the accuracy of eyewitness testimony*.” However, in their study, only 61% of these experts deemed the statement reliable enough to present in court (see also Kassin et al., 2001). Importantly, the opinions of eyewitness memory experts and fundamental memory experts diverged widely regarding stress effects during encoding. While 78% of fundamental memory experts agreed that “*Experiencing stress during an event (i.e., at encoding) enhances memory for that event,”* only 32% of eyewitness experts did, highlighting the lack of consensus even amongst memory experts.

Pezdek and Reisberg acknowledged the complexity of the stress-memory relationship by referring to a meta-analysis (Shields et al., 2017) that suggests that encoding stress may enhance memory for stressor-relevant information when there is no or little delay between encoding and the stressor. However, Pezdek and Reisberg concluded that these conditions for encoding stress improving memory were limited to a “narrow focus” (p. 145) and implied that situations where stress impaired memory were more common. In reality, though, eyewitnesses frequently experience stress and encoding simultaneously, and the type of to-be-remembered information is often directly related to the stressor in a crime situation. These factors are in line with the moderating conditions for memory enhancements within the meta-analytic findings. Both of these factors also align with neurobiological theories and findings of many acute stress studies in the fundamental memory field suggesting memory enhancements (e.g., Joëls et al., 2006; Marr et al., 2021b, for a review).

To provide evidence against Myth #2, Pezdek and Reisberg cited findings from the eyewitness memory field suggesting that encoding stress impairs memory. However, this past work suffers from serious methodological limitations (Sauerland et al., 2016; Marr et al., 2021b). Many eyewitness studies conduct the memory retrieval test within minutes after the stressor/encoding phase (e.g., Brigham et al., 1983; Stanny and Johnson, 2000; Davis et al., 2019; Pezdek et al., 2020; Price et al., 2022). Because stress has an opposite effect on memory encoding (i.e., enhancing) and retrieval (i.e., impairing), this lack of sufficient retention interval obstructs any conclusions about the effects of encoding stress effects on memory.^2^ Additionally, the majority of eyewitness studies (e.g., Davis et al., 2019; most studies in Deffenbacher et al., 2004; Pezdek et al., 2020) have relied on self-reports of stress rather than more objective, physiological measures, such as blood pressure or cortisol. Self-reported measures are valuable for application to real life, where physiological, objective measures are often unobtainable. However, for experimental lab studies, this measurement issue raises the question of whether the effect of encoding *stress* on memory was actually captured—or merely an effect of *arousal* (or a number of other cognitive phenomena). Researchers should strive to ensure that stress is properly induced and verified by using objective measures wherever possible, alongside self-report measures (cf. Shields et al., 2017; Marr et al., 2021b). If physiological measures of stress cannot be included, researchers should be cautious in using the term “stress” with respect to its effects on memory without noting this limitation. This care in terminology is particularly important for eyewitness studies, which often involve complex scenarios that likely produce many other effects, including the impact of arousal, divided attention, perceptual phenomena, or cognitive load. More studies examining links between self-reported levels of stress and physiological states of stress would be helpful for improving the construct validity of self-report measures, and in turn, will improve application to reality (e.g., Weber et al., 2022).

Pezdek and Reisberg (2022) also discredited the (ecological) validity of stress induced by the Trier Social Stress Test (TSST; see footnote 2 on p. 146). Dozens of studies collecting physiological measures alongside self-reports and recent meta-analyses have confirmed the validity of the TSST for inducing a full stress response (e.g., Goodman et al., 2017; Seddon et al., 2020; Gu et al., 2022). In contrast, it is currently unclear how “stressors” used in many eyewitness studies score on these dimensions (e.g., emotional pictures, violent videos, false fire alarms, Joëls et al., 2006; Marr et al., 2021b). Given that to-be-remembered materials that are directly related to the stressor elicit stronger effects (Shields et al., 2017), this should motivate eyewitness memory and stress researchers to collaborate in designing studies that combine the best of both fields to study the effects of encoding stress on memory (cf. Marr et al., 2021a). However, the fact that stress elicited in the TSST is not directly related to the to-be-remembered material does not justify discarding all findings that derive from its use—or effectively throwing the baby out with the bath water.

## Conclusion and implications

We conclude that the empirical research base to date does not allow for any strong conclusions about the effect of encoding stress on memory. Rather, whether acute stress impairs, enhances, or does not reliably affect memory performance is dependent on many moderators, most of which still need to be more thoroughly investigated in future research (Marr et al., 2021b). Eyewitness reports from those who have been through a stressful experience should not be immediately accepted *or* discounted without examining the surrounding context and keeping the findings from *both* the eyewitness and fundamental memory fields in mind.

Future research on this topic will provide a clearer understanding of the factors that critically contribute to the relationship between stress and memory and the direction of that relationship. In the meantime, however, it is important to acknowledge the existing shades of gray when discussing stress effects on memory, particularly in applied legal settings. That being said, certain sub-topics relevant to the stress-memory relationship in eyewitness settings do show greater expert consensus than others (e.g., those related to stress severity and detail type; Marr et al., 2021a). Additionally, strong expert consensus exists regarding the inaccuracy of certain widespread layperson beliefs, including ideas that police officers are less influenced by acute stress or that stressful experiences can cause memory repression. These incorrect beliefs can and should be countered where relevant by expert witnesses in court.

## Author contributions

CM drafted the commentary. All other authors provided critical feedback and contributed to the development and finalization of the commentary.

## Footnotes

^1^In this commentary—like Pezdek and Reisberg—we focus on *acute* rather than *chronic* stress, where a stressor is long-lasting and continuous. Though not discussed further in this commentary, future studies should consider the unique effects of chronic stress on memory performance (Wolf, 2008; Finsterwald and Alberini, 2014) and the interaction between acute and chronic stress, which may be relevant to legal settings when considering ongoing or repeated events such as family violence crimes.

^2^Note that this type of research is important in its own right—if the research question aims to examine how acute stress may affect immediate memory performance (e.g., Krix et al., 2016). However, if researchers specifically aim to examine effects of encoding stress, a retention interval of at least 24 h is needed to properly separate the encoding and retrieval memory stages due to the lengthy timeline of a physiological stress response (Joëls and Baram, 2009).
